# Hyperprogression in Pediatric Melanoma Metastatic to the Breast Treated with a Checkpoint Inhibitor

**DOI:** 10.7759/cureus.3859

**Published:** 2019-01-09

**Authors:** Laura Bernal Vaca, Sara D Mendoza, Juan C Vergel, Xavier Rueda, Ricardo Bruges

**Affiliations:** 1 Oncology, Instituto Nacional De Cancerología, Bogotá, COL; 2 Oncology, Instituto Nacional De Cancerologia, Bogotá, COL; 3 Surgery, Instituto Nacional De Cancerologia, Bogotá, COL; 4 Dermatology, Instituto Nacional De Cancerologia, Bogotá, COL

**Keywords:** melanoma, pediatric, immunotherapy, progression, metastasectomy

## Abstract

Metastatic melanomas in the pediatric population are rare, but they have been appearing more frequently. Unfortunately, little is known about the differences in the biology and therapeutic implications of pediatric metastatic melanomas when compared to those found in adults. Herein, we have presented the case of a 13-year-old girl with a stage IIID malignant melanoma arising from a congenital nevus. This patient underwent surgical management, and she received adjuvant interferon therapy; however, this treatment was incomplete due to a grade 3 transaminase elevation and the early recurrence of the disease. An isolated metastasis to the breast was documented, and a mastectomy was performed. Soon afterward, low-volume lung metastases developed, and she was treated with nivolumab. After two treatment cycles, the disease continued to develop in a hyperprogressive manner.

Advances in the characterization and understanding of pediatric melanomas are needed, as well as experience in the management of new therapies in these cases, which would help clarify the extent to which we can extrapolate the data obtained from the adult population. Therapeutic interventions in melanoma cases are evolving rapidly, and the role of metastasectomies in the era of immunotherapy and BRAF and MEK-targeted therapies is largely unknown. Moreover, the identification of risk factors for the development of hyperprogression and its underlying mechanisms are also warranted.

## Introduction

Melanoma is a disease predominantly affecting adults, with an uncommon presentation in the pediatric population; however, approximately 2% of all melanomas occur in patients under the age of 20 years and 0.4% occur in the prepubertal age group [1-2]. The pediatric melanoma incidence has been increasing at an alarming rate of 2% per year [3].

Due to their low incidence in this population, melanomas are rarely suspected. They are frequently diagnosed at advanced stages that are associated with unfavorable prognoses. Additionally, melanomas tend to grow faster in the pediatric population than in adults, and they develop early metastases group [1-2]. The pediatric melanoma risk factors include congenital nevi, the total body nevus count, dysplastic nevi, and familial atypical multiple mole melanoma syndrome. The presence of congenital nevi is associated with a relative risk of 34 with more than 100 large nevi (greater than 5 mm in diameter) and a relative risk of 15 in children with more than one [3]. This is an association of great importance for diagnostic suspicion.

Approximately 22% of patients with pediatric melanomas have non-modifiable risk factors, such as fair skin, genetic susceptibility, and family history. In these cases, prevention is an essential component, because the accumulation of mutations induced by ultraviolet rays plays a critical role in the development of melanomas. Moreover, it has been reported that up to 60% of the children under 10 years old who are diagnosed with melanomas do not exhibit the most commonly suspected features [2-3]. Specific criteria have been proposed for this population, as follows: A = amelanotic, B = bleeding/bump (swelling), C = color uniformity, D = *de novo*/any diameter, and E = evolution [2-3]. Therefore, a dermoscopic examination is a valuable tool for detecting and monitoring pediatric skin lesions. Unfortunately, no data have been published on the frequency and prognostic value of the BRAF mutation in the pediatric population.

## Case presentation

**Figure 1:**
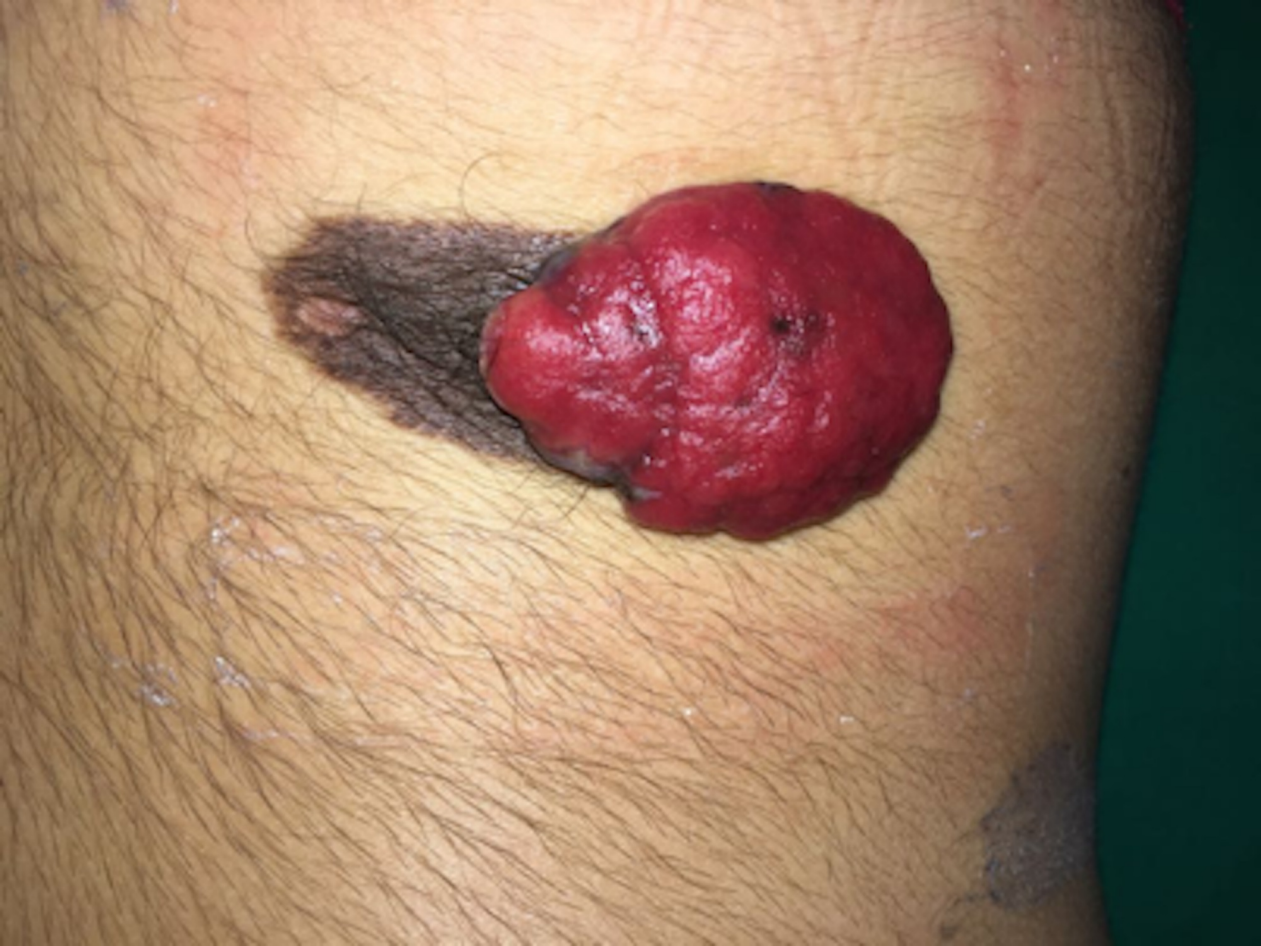
Primary tumor localized in the left lumbosacral region: congenital nevus and malignant melanoma

A 13-year-old phototype III female patient presented to our institution with a congenital nevus on her left lumbosacral region with a large diameter of 8 cm. At the time of the consultation, it had been one year since the appearance of a rapidly growing exophytic lesion on this congenital nevus, which exhibited color changes, edge irregularity, bleeding, and occasional pain. The physical examination of this patient revealed a 6 x 4-cm erythematous tumor with active bleeding on top of an 8.5 x 3-cm dark brown plate (Figure 1).

**Figure 2:**
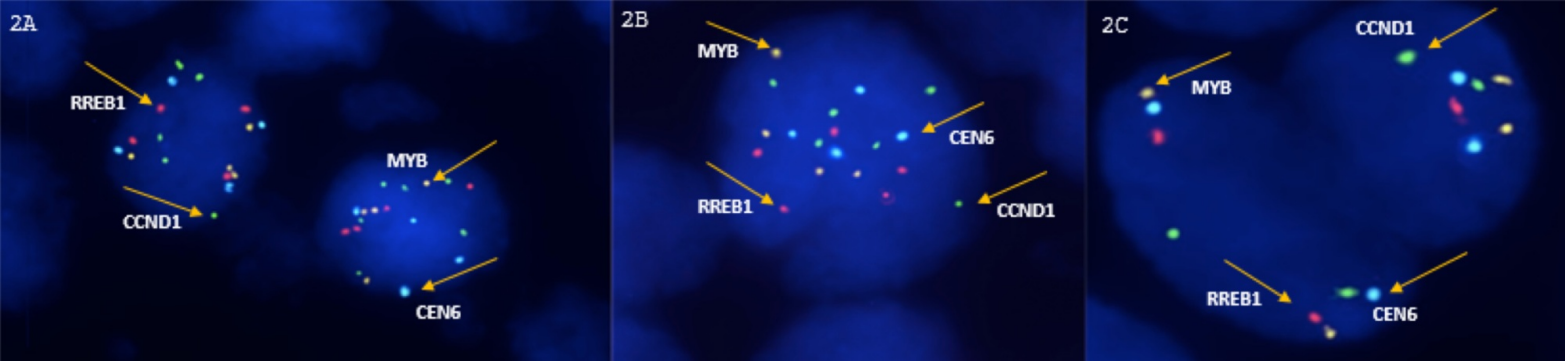
Fluorescent in situ hybridization (FISH) for RREB1, MYB, and CCND1 2A and 2B: Invasive melanoma: positive FISH (higher number of signals from CCND1 and RREB1 probes); 2C. Benign nevus: negative FISH.

The total body photography and digital dermoscopy (FotoFinder Systems, Inc., Columbia, MD, USA) documented more than 20 additional melanocytic lesions. A biopsy of the lumbosacral lesion was obtained, and the histopathological results showed a superficial spreading malignant epithelioid melanoma. The fluorescence in situ hybridization (FISH) results for the RREB1, MYB, and CCND1 genes (common molecular alterations in malignant melanomas) were positive for the tissue obtained from the new exophytic lesion and negative for the congenital nevus tissue (Figure 2).

**Figure 3:**
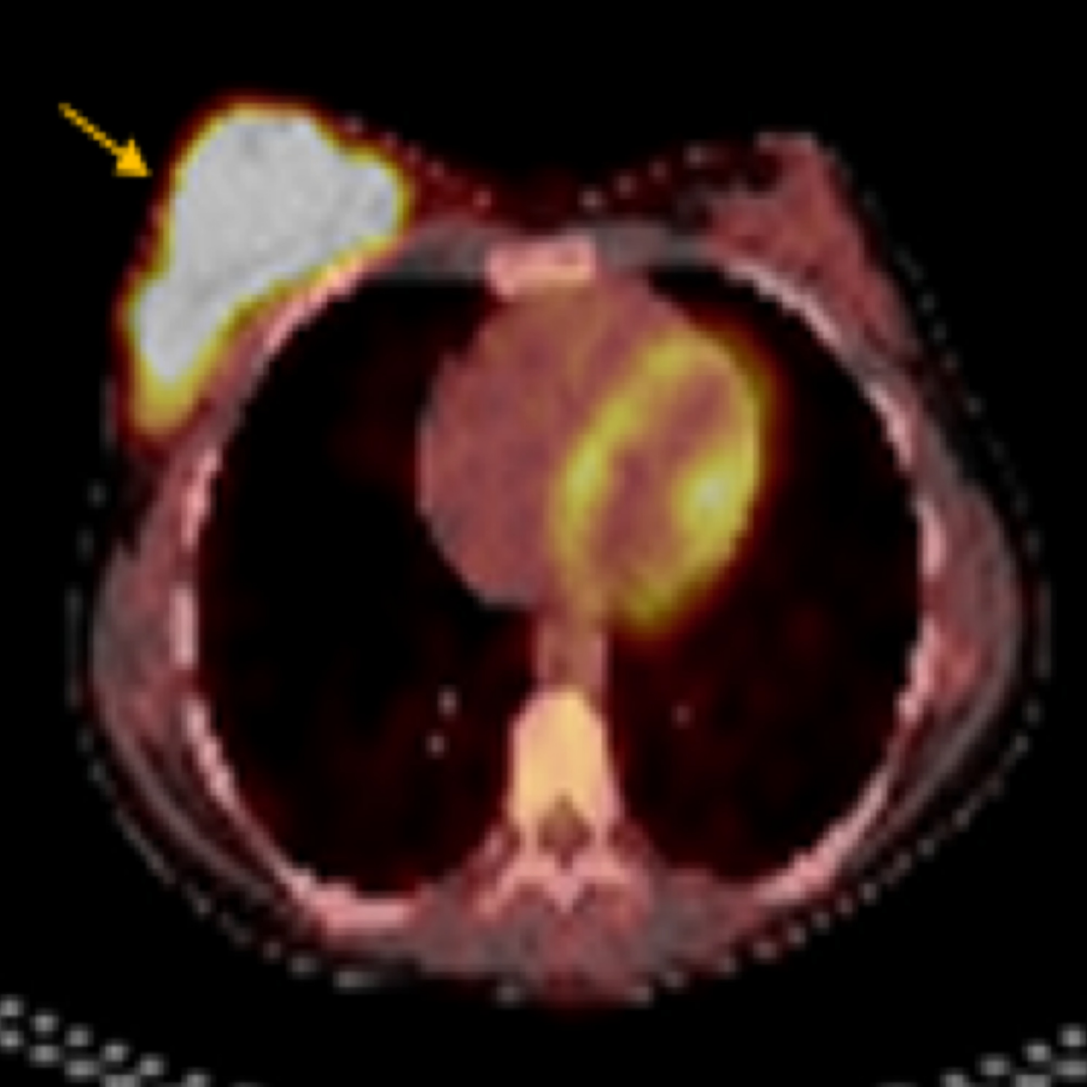
PET-CT: Isolated metastasis of melanoma to the right breast (12 SUV) PET-CT: positron emission tomography-computed tomography

This patient underwent a wide local excision, sentinel lymph node biopsy, and flap reconstruction. The pathological results were as follows: a Breslow’s tumor thickness of 13 mm, Clark Level V, extensive ulceration, mitoses of 10/mm^2^, negative margins, and sentinel lymph nodes with extensive metastatic involvement. A lymphadenectomy was also performed, and 20 lymph nodes were obtained, six of which were positive. Based on the above-mentioned results, this patient was diagnosed with a stage IIID melanoma or clinicopathologically, T4bN3aM0, according to the American Joint Committee on Cancer (AJCC) Cancer Staging Manual, 8th edition. The polymerase chain reaction (PCR) showed no BRAF mutations. She was given interferon-α-2b as an adjuvant treatment, but it caused toxicity during the first week of the induction phase, with a grade 3 transaminase elevation. At that point, the treatment was withheld, and the patient was voluntarily lost to follow-up for four months. When she returned for treatment, the interferon was resumed at the maintenance phase for two additional months of treatment. Then, she presented to the emergency room with a rapidly growing mass in her right breast. A biopsy was obtained, which confirmed the diagnosis of metastatic melanoma. Positron emission tomography-computed tomography (PET-CT) was used to identify the mass, and it had completely replaced the breast tissue with a standardized uptake value of 12 (Figure 3). There was no evidence of metastases in any other site.

A metastasectomy was performed (right mastectomy plus axillary lymph node dissection), and an 11-cm tumor was obtained with a 1-mm margin. Three of the 28 lymph nodes that were removed were positive for metastatic involvement. Postmetastasectomy, with no approved active therapy, this patient was closely monitored. The subsequent tomographies (less than two months after surgery) showed multiple metastatic bilateral pulmonary nodules and right axillary matted lymph nodes. This conglomerate was noticeably painful, and so radiotherapy was administered with a palliative intention (total dose of 30 Gy).

**Figure 4:**
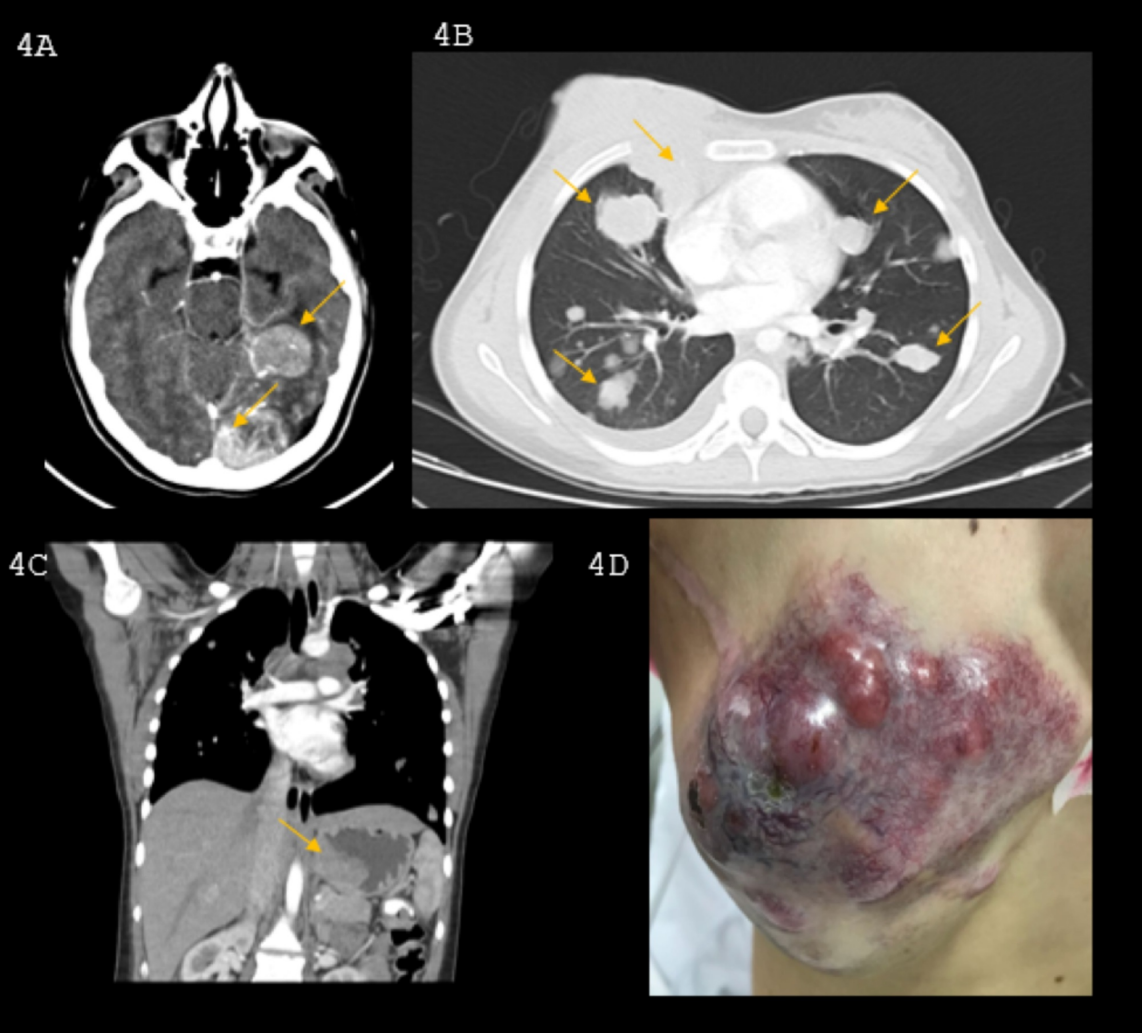
Hyperprogression after immunotherapy A. Central nervous system: left temporal and occipital compromise with marked vasogenic edema of the adjacent parenchyma; B. Lung metastases and mediastinal involvement at the prevascular level, adjacent to the right atrium, descending aorta, and left ventricle; C. Mass in the gastric fundus; D. Recurrence of the breast mass.

In a multidisciplinary meeting, with the Ethics Committee’s approval, systemic nivolumab therapy was chosen as a palliative treatment, based on the extrapolation of evidence from adult cases. This patient tolerated the treatment well; however, after two cycles, she exhibited gastrointestinal bleeding. An upper endoscopic evaluation showed a 6-cm gastric exophytic mass, and a biopsy confirmed the diagnosis of metastatic melanoma. This patient was reassessed, and an explosive progression of the disease was found, with a 50% growth in her lung lesions and the appearance of new hepatic, retroperitoneal, and mediastinal masses of up to 4 cm. Some of these were adjacent to the right atrium, descending aorta, and left ventricle. Additionally, metastatic lesions had developed in the central nervous system (infundibulum and pituitary gland) and an 8.5-cm ulcerated mass reappeared on the thoracic wall at the location of the previous mastectomy (Figure 4).

We considered this as hyper-progression, and a second line with carboplatin and paclitaxel was proposed. Whole brain palliative radiotherapy was carried out, and gastrointestinal bleeding became difficult to control, with chemotherapy having little effect. Hence, we offered palliative radiation to the gastric mass and chest wall mass to alleviate symptoms, with limited response. Her performance quickly deteriorated over the next few weeks, until treatment was suspended. The patient was offered the best support care possible before she passed away. 

## Discussion

Breast metastasis from extramammary tumors comprises 2% of all malignant breast tumors. Twenty percent are caused by melanoma, which is more frequent in young patients [4-5]. The most common primary location of metastatic melanoma of breast is the chest wall and upper limbs [5-6]. On a radiological evaluation, these lesions usually appear as nodular, hypoechoic, or solitary, with circumscribed borders, well-defined margins, and variable axillary node involvement (25% to 80% of patients). They can clinically and radiologically mimic primary tumors; hence, relevant studies should be conducted to determine the origin of metastasis and the presence of the disease in other areas. In some cases, surgical management is to be offered. Surgical management has to be adjusted to the disease prognosis, and the objective of the intervention must be established to avoid radical surgery in a palliative scenario [7-9].

Evidence for obtaining an overall survival benefit by performing a metastasectomy in melanoma comes from retrospective and phase II studies. Appropriate patient selection in a multidisciplinary context is a key factor, taking into account disease-free intervals, time of tumor doubling, number of lesions or compromised organs, response to previous treatments, and performance status [10-11]. The intention is curative but the rate of post-metastasectomy recurrences are high, given the frequency of micrometastatic diseases [12-14]. In a prospective, multicenter, phase II study of the Southwest Oncology Group (SWOG), a complete resection of metastasis was achieved in 64 patients where the average progression-free survival was five months, and the overall survival was 21 months, with survival rates at three and four years on 36% and 31%, respectively. Nevertheless, late relapses continued to be observed after this time [12]. A review of the database in Surveillance, Epidemiology and End Results (SEER) collected data from 4,229 patients with stage IV melanoma between 1988 and 2006 and found better overall survival in patients undergoing metastasectomies (average of 12 versus five months in non-surgical management, at five years, 16% versus 7%). In M1a disease, the benefits were more pronounced at an average of 14 versus six months, and at five years 20% versus 9% [14]. In an MSLT-1 study, patients who underwent metastatic recurrences were analyzed, and among those taken to metastasectomy, a survival rate of up to 45% at four years, median 16 months was documented [15]. Traditionally, no therapy was shown to be able to modify post-metastasectomy outcomes; hence, we proceeded to observation after surgery. Recently, CheckMate-238 of nivolumab in adjuvant treatment included 82 patients with a resected stage IV disease, mostly M1a. The primary outcome favored intervention for the entire population, and for this subgroup, a nonsignificant benefit was obtained with an absolute benefit in recurrence-free survival of 5% at 12 months, 63 versus 58%, median not reached versus 16.8 months, HR 0.70 (0.45, 1.10). This analysis has all the well-known caveats of subgroup analysis, meaning we require more data (e.g. overall survival effect) before incorporating this as a standard of care. Nonetheless, this intervention has been widely adopted, considering the poor prognosis of the disease and the absence of other effective therapies in this scenario [16].

Hyperprogression has been defined as disease progression based on RECIST (Response Evaluation Criteria In Solid Tumors), with a two-fold increase in the tumor growth rate during treatment compared with tumor growth rates before treatment. Its frequency seems to be lower in melanoma than in other cancers, such as lung, head, and neck cancer. Predictive factors are poorly established in patients treated with anti-programmed cell death protein 1 (PD-1) immunotherapy [17]. The acceleration of tumor growth has been attributed to the exacerbation of oncogenic signaling. Depending on genetic alterations of the tumor cells, it is possible that blocking PD-1 affects alternative signaling networks and potentiates growth and/or tumorigenesis.

Possible identified risk factors include accelerated tumor growth prior to the beginning of treatment (and tumor doubling time), the number of metastatic sites, and the sum of the longest diameters of target lesions. However, none have shown consistent statistical significance across studies. Risk factors have been identified more frequently in elderly patients (older than 65 years), which raises the hypothesis that the development of hyper-progression may be closely related to basal immunological conditions (for example, immunosenescence, expression of co-stimulatory/co-inhibitory proteins in T cells, or higher concentrations of inflammatory cytokines). Specific genetic alterations have been identified in patients developing hyper-progression, such as MDM2, or in genes involved in the interferon pathway. [17]

The question is open: what would be the features of the interaction between a child's or prepubertal’s immune system with an anti-PD1 therapy?

The efficacy and safety of immunotherapy in pediatric patients still need to be clarified. Multicenter phase I and II studies with checkpoint inhibitors have been carried out in this population, which were presented at the 2017 American Society of Clinical Oncology (ASCO) Conference (iMATRIX study with Atezolizumab, KEYNOTE 051 study with Pembrolizumab and AVDL study 1412 with Nivolumab plus Ipilimumab). These findings included very few patients with melanoma [18-20]. The safety population was acceptable, although a higher incidence of some grade 3 and 4 treatment-related adverse events was identified, including hypertransaminasemia, fatigue, vomiting, and abdominal pain. Efficacy seems to be somewhat lower than in adults, especially in monotherapy, but a combination of nivolumab + ipilimumab often led to unacceptable toxicity. The expression of programmed death-ligand 1 (PD-L1) was low and infrequent in these patients; hence, there is an urgent need for other biomarkers in this population. 

Both pembrolizumab and nivolumab are approved by the US Food and Drug Administration in the pediatric population (over 12 years old) for tumors with microsatellite instability based on data extrapolated from adults.

## Conclusions

To our knowledge, this is the first reported case of hyper-progression to immunotherapy in melanoma in the pediatric population. Metastatic melanoma in the pediatric population is a rare condition that is consistently becoming more frequent. A joint effort is required to make progress in the characterization and understanding of this disease and its behavior in order to identify the biological differences and therapeutic implications in comparison with adult melanoma. We need to gain and share experiences in the management of new therapies for this population, clarifying to what extent we can extrapolate the data obtained from adults. Therapeutic interventions in melanoma are evolving rapidly, leaving behind questions about the role of metastasectomies in the era of immunotherapy and BRAF-MEK-targeted therapy and how we can better identify those patients who will develop hyper-progression.
